# Supplementary material for: Inhibition of Cytomegalovirus by Pentacta pygmaea Fucosylated Chondroitin Sulfate Depends on Its Molecular Weight
Source: Viruses. 2023 Mar 28;15(4):859. doi: 10.3390/v15040859 (PMC10142442; doi:10.3390/v15040859)

Figure S1. Anti-HCMV activity of native PpFucCS.

The native PpFucCS was assayed for its potential to inhibit the HCMV production in HFFs (MOI 3) by plaque assay in duplicates of two independent experiments. The normalized values from the assay were analyzed by nonlinear regression to fit a dose-response curve by the least squares method considering each value as an individual point. The plotted curve shows the percentage of HCMV inhibition in a (log) concentration-dependent manner.

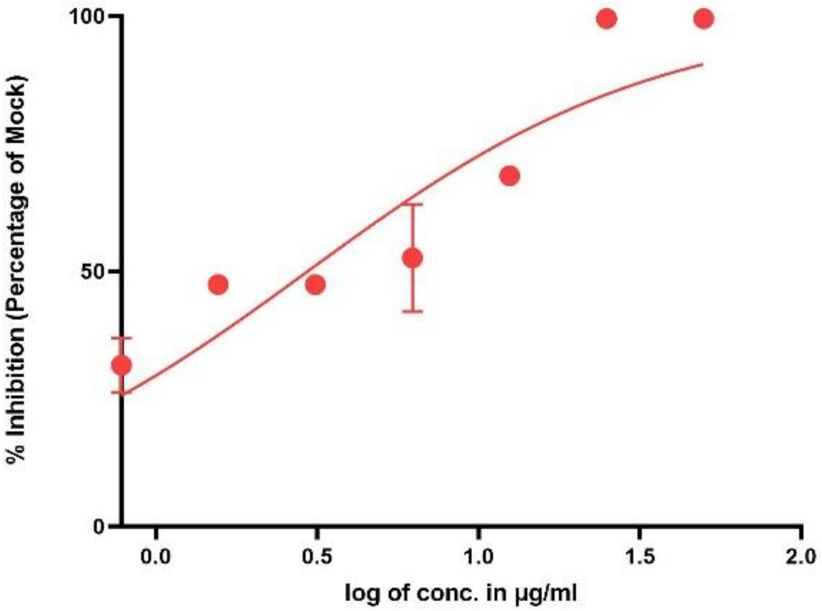

Figure S2. Effect of PpFucCS on the cytotoxicity of HFF cells.

HFFs were treated with native PpFucCS (500 μg/ml) and unfractionated heparin (UFH) (500 μg/ml) for 5 days and cell viability was assessed using Trypan Blue exclusion assay. Bars in the plot represent the following: mock-treated (grey), PpFucCS-treated (red with black pattern), and UFH-treated (black with grey pattern). The experiment was repeated two times to confirm reproducibility. The standard error of the mean was plotted as error bars. The ***, **, *, and ns indicate p-value < 0.001, between 0.001 and 0.01, between 0.01 and 0.05, and ≥ 0.05, respectively.

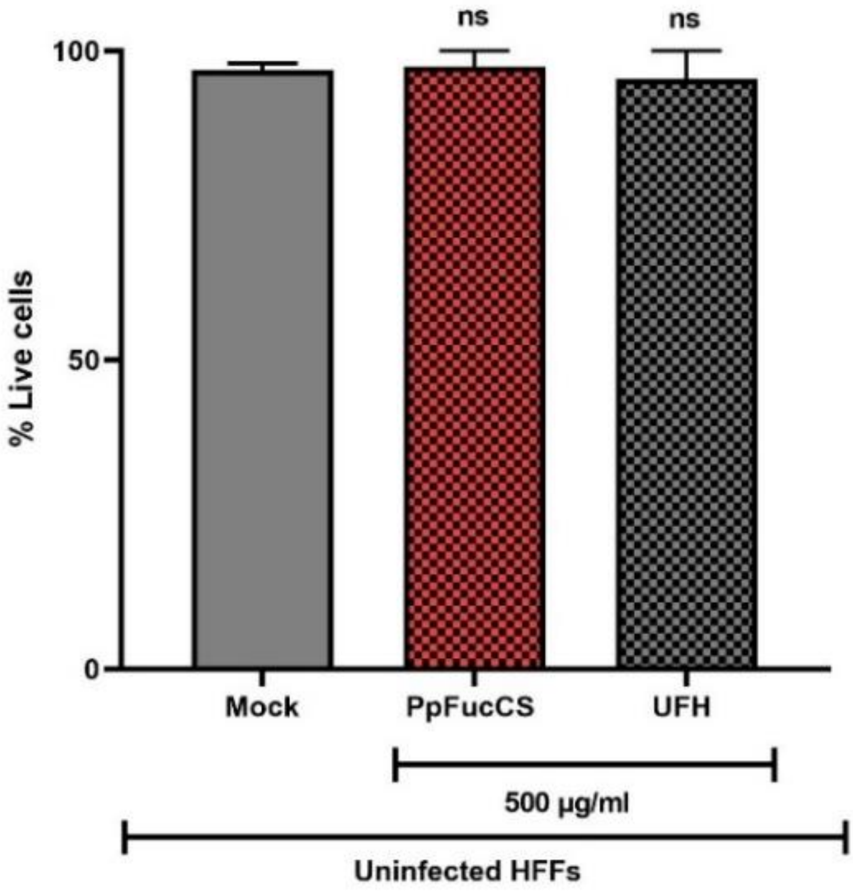

Figure S3. Assessing the cytotoxicity of PpFucCS by quantifying the ATP release.

HFFs were treated with native PpFucCS at 50 μg/ml and at 500 μg/ml for 5 days in duplicates and cell viability was assessed using bioluminescent ATP assay. Bars in the plot represent the following: mock-treated (grey), PpFucCS-treated at 500 μg/ml (red with black pattern), PpFucCS-treated at 50 μg/ml (red), UFH-treated at 500 μg/ml (black with grey pattern), and UFH-treated at 50 μg/ml (black). The experiment was repeated two times to confirm reproducibility. The standard error of the mean was plotted as error bars. The ***, **, *, and ns indicate p-value < 0.001, between 0.001 and 0.01, between 0.01 and 0.05, and ≥ 0.05, respectively.

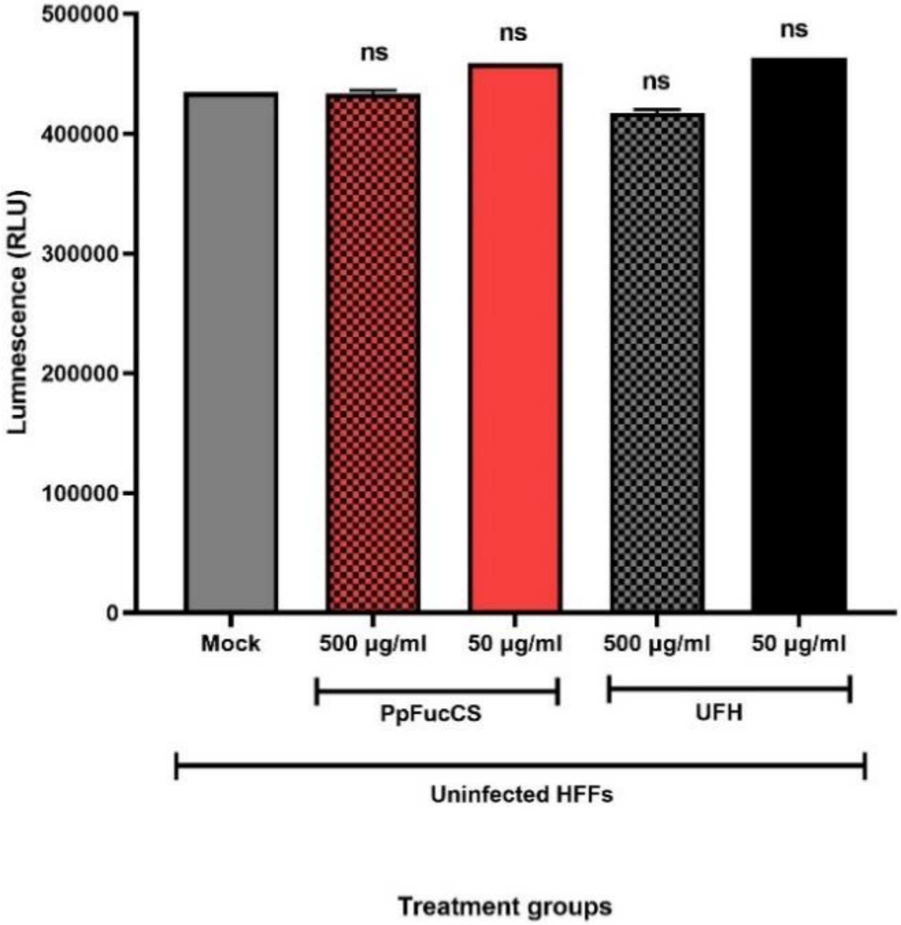

Supplement: Supplementary file 1 [file viruses-15-00859-s001.zip › viruses-2130625-supplementary.pdf]
